# Renal angiomyolipoma during pregnancy: Case report and literature review

**DOI:** 10.4274/tjod.32848

**Published:** 2015-06-15

**Authors:** Cihan Çetin, Selim Büyükkurt, Cansun Demir, Cüneyt Evrüke

**Affiliations:** 1 Çukurova University Faculty of Medicine, Department of Obstetrics and Gynecology, Adana, Turkey

**Keywords:** Angiomyolipoma, nephrectomy, Pregnancy

## Abstract

Renal angiomyolipoma is a rare tumor that can be either sporadic or found together with tuberous sclerosis or pulmonary lymphangioleiomyomatosis. These tumors are hormone sensitive and therefore tend to grow during pregnancy and their main complication is the risk of rupture. Optimal management is still controversial because there are very few cases reported in the literature. We expect that the case of our patient, who delivered her baby vaginally at 36 weeks of gestation and underwent definitive treatment (nephrectomy) thereafter, to further enhance the knowledge about the management of these rare tumors during pregnancy.

## INTRODUCTION

Renal angiomyolipoma (RA) are rare tumors with an incidence of 0.3% in the general population and are even rarer among women who are pregnant^(1)^. These tumors arise from epithelioid cells around blood vessels, can be sporadic, but may also be associated with tuberous sclerosis complex (TSC) or pulmonary lymphangioleiomyomatosis (LAM)^(2)^. Although symptoms like hematuria or flank pain may occur, they are more frequently detected incidentally during imaging for unrelated reasons.

These hormone-sensitive tumors (estrogen, progesterone) may rupture during pregnancy due to elevated levels of estrogen and progesterone; most of the cases in the literature ruptured during pregnancy. We present a patient who was pregnant whose renal mass was diagnosed during pregnancy. The patient was managed conservatively in the antenatal period without any complication, and then given definitive treatment after the birth. We reviewed the literature about cases of RA that have been managed during pregnancy.

## CASE REPORT

**Figure 1:**
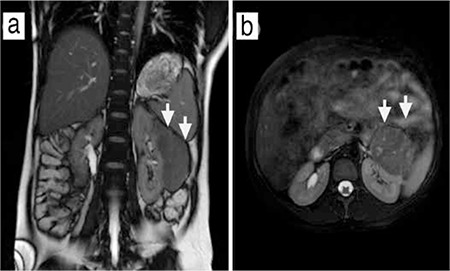
Magnetic resonance imaging of the renal tumor a) coronal section, b) axial section (arrow indicating the tumor)

**Figure 2:**
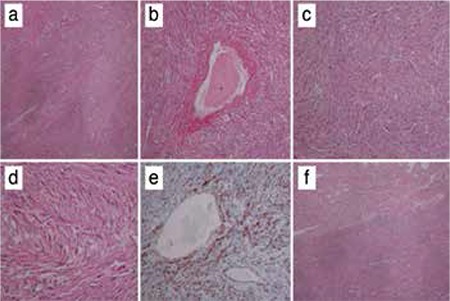
a) Hematoxylin&Eosin stain under x40 magnification, spindle cells in fascicular pattern, starting from the perivascular area, b) x100 magnification, c) x200 magnification, smooth muscle cells component, d) x400 magnification, e) x100 magnification, HMB-45 positivity in immunohistochemistry, f) x40 magnification, tumor area in the upper part, normal renal tissue below

A primigravida women aged 26 years was referred to our clinic during her 34^th^ week of gestation with left flank pain. Her medical history was not significant for any disease. The patient had no costovertebral angle tenderness on either side. Her urine analysis revealed pyuria but not hematuria. A urinary tract infection was treated with oral ampicillin. The patient’s vital signs were stable and there was no fever. Fetal biometric measures were compatible with 34 weeks and the amniotic fluid index was normal on ultrasound evaluation. A urinary system ultrasound revealed a 9.9x5.8-cm hypoechoic solid lesion on the left kidney that had distorted the renal calices. Magnetic resonance imaging (MRI) results showed a lobulated, heterogeneously contrasted 11x6.5x5.5-cm soft tissue mass lesion arising from the middle segment of the left kidney that contained necrotic areas (Figure 1). Renal function tests were within the normal range. The patient delivered vaginally after cervical ripening with dinoprostone and induction with 4 oxytocine, consecutively, at 36 weeks of gestation due to the patient’s anxiety about the risk of tumor rupture. The neonate was a 2430 g girl with 1^st^/5^th^ min Apgar scores 9/10, respectively. The neonate was discharged without any problems. Two weeks after the delivery, the patient underwent laparoscopic a radical left nephrectomy. Pathologic evaluation of the specimen showed epitheloid angiomyolipoma with intact tumor-free borders around the mass (Figure 2). After being discharged from hospital, the patient had no complaints in the third month after the operation. The patient had normal neurologic, pulmonary, and skin examination, by which we excluded TSC and LAM.

## DISCUSSION

More than 25% of RA’s carry estrogen and progesterone receptors like in pulmonary LAM^(3,4)^. Therefore, they are thought to grow during pregnancy or with oral contraceptive use. Symptoms may occur during these periods in previously asymptomatic patients. There are few case reports about RA’s during pregnancy in the literature and these are summarized in Table 1.

Although most angiomyolipomas are located in the kidneys, they can also be diagnosed in other organs like liver, spleen or uterus as they arise from epitheloid cells around blood vessels^(5)^. Some cases can present with sudden abdominal or flank pain and with symptoms of hypovolemic shock, which suggests rupture of the tumor into the retroperitoneal area; however, they are frequently asymptomatic. In cases of rupture, hemodynamic stability is of critical importance regarding the selection of optimal treatment strategy. For hemodynamically unstable patients, emergency surgery or arterial embolization (if available) are the main options of treatment. For asymptomatic patients, a conservative approach may be chosen, especially during pregnancy^(5)^. For these patients, definitive treatment may be postponed until the postpartum period^(6)^. Similarly, we delayed the definitive treatment of the tumor to the postpartum period. However, there is as yet no consensus in the literature as to how long the conservative approach is suitable. Close follow-up of patients who are pregnant may be preferred because of the high risk of rupture.

Most of the cases during pregnancy reported in the literature ruptured during pregnancy (21/26, 81%). To the best of our knowledge, only four (15%) of the 26 cases reported (including ours) have not ruptured during pregnancy (Table 1). These were managed with nephrectomy during or after pregnancy^(7,8,9)^.

The mean age and mean gestational age of the patients in the literature at the time of diagnosis was 31.4 years and 27.7 weeks, respectively. Twenty-one of these cases (81%) ruptured (Table 1). The average size of the tumors measured with ultrasound or MRI was 10.1 cm and tumor size did not correlate with the risk of rupture. The tumor in our case was 11 cm and rupture did not occur tumor, whereas in dos Santos et al.’s case, rupture was reported with a tumor that measured 5 cm^(11)^. Therefore, estimation of the risk of rupture using only tumor size would not be accurate.

Most patients with RA in the literature delivered their babies via cesarean section (15/26, 56%), whereas only 5 (19%) were delivered vaginally (Table 1). However, vaginal delivery can be considered as a safe approach for these patients. Mode of delivery for these patients should be decided based on obstetric indications, because cesarean section does not reduce the risk of rupture. Vacuum extraction can also be an alternative option for these patients in order to shorten the second stage of the labor. In our case, we did not need vacuum extraction due to precipitous labor.

Current treatment options for RA include partial/total nephrectomy (open or laparoscopic), cryoablation, radiofrequency ablation, or arterial embolization^(3)^. Fourteen (54%) of the patients reported in the literature needed nephrectomy, whereas 12 (46%) were treated conservatively with or without arterial embolization (Table 1). Embolization can also be performed after 12 weeks of gestation with minimal fetal radiation exposure^(3)^. This can be performed using devices such as coils, gelfoam, or polyvinylalcohol^(3)^. The main complications of embolization are Post-embolization syndrome (POS) (inflammation, fever, leukocytosis, and flank pain) and liquefactive necrosis^(3)^. POS generally resolves spontaneously, whereas percutaneous drainage might be needed for liquefactive necrosis^(3)^. In contrast to embolization or ablation, surgery has the important advantage of allowing for pathologic evaluation for a definitive diagnosis. Differential diagnosis of these lesions includes renal cell carcinoma, oncocytoma, and metastatic lesions from primary tumors elsewhere. Radiographic features can usually distinguish these from each other. For those patients in whom radiographic evaluation is not enough for definitive diagnosis as in our case, either biopsy or nephrectomy (as a definitive treatment) can be performed.

In conclusion, renal angiomyolipomas are rare tumors but physicians who encounter renal masses during pregnancy should keep these in mind, because they can grow and be symptomatic for the first time during pregnancy. MRI is usually enough for diagnosis, but for uncertain cases, biopsy or surgery can be performed. Treatment strategies should be individualized due to insufficient data in the literature to support any one in particular. More experience with these strategies is needed before an optimal treatment method can be recommended. It is our hope that the distinctive features of our case will further enhance the knowledge of the management of these rare tumors during pregnancy.

## Figures and Tables

**Table 1:**
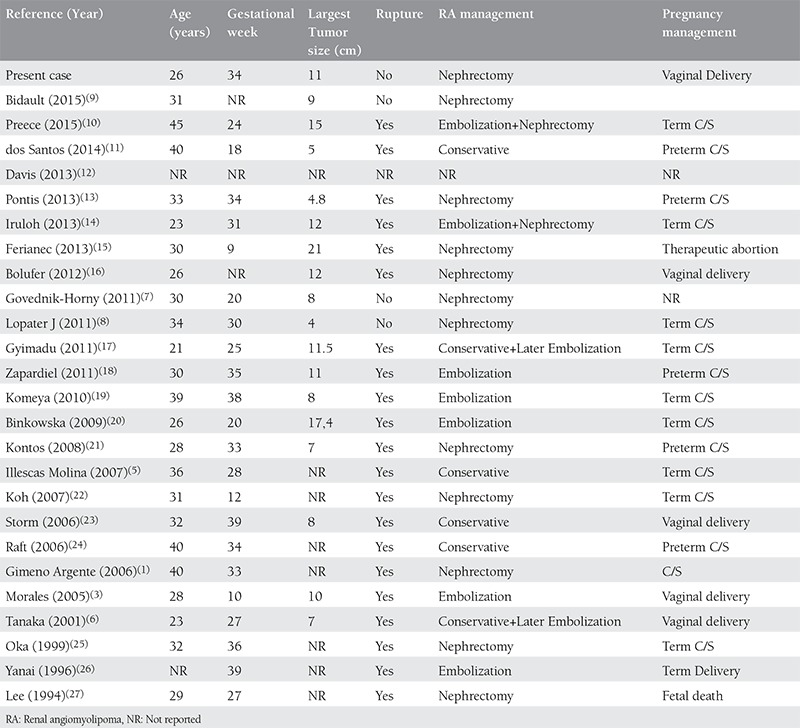
Literature review of renal angiomyolipoma cases during pregnancy
